# Complementary neural correlates of motivation in dopaminergic and noradrenergic neurons of monkeys

**DOI:** 10.3389/fnbeh.2012.00040

**Published:** 2012-07-17

**Authors:** Sebastien Bouret, Sabrina Ravel, Barry J. Richmond

**Affiliations:** ^1^Laboratory of Neuropsychology, NIMH/NIH, BethesdaMD, USA; ^2^Team Motivation Brain and Behavior, ICM/CRICMParis, France; ^3^Institut de Neurosciences de la Timone, CNRS, Aix-Marseille UniversitéMarseille, France

**Keywords:** neuromodulation, reward, locus coeruleus

## Abstract

Rewards have many influences on learning, decision-making, and performance. All seem to rely on complementary actions of two closely related catecholaminergic neuromodulators, dopamine (DA), and noradrenaline (NA). We compared single unit activity of dopaminergic neurons of the substantia nigra pars compacta (SNc) and noradrenergic neurons of the locus coeruleus (LC) in monkeys performing a reward schedule task. Their motivation, indexed using operant performance, increased as they progressed through schedules ending in reward delivery. The responses of dopaminergic and noradrenergic neurons around the time of major task events, visual cues predicting trial outcome and operant action to complete a trial were similar in that they occurred at the same time. They were also similar in that they both responded most strongly to the first cues in schedules, which are the most informative cues. The neuronal responses around the time of the monkeys' actions were different, in that the response intensity profiles changed in opposite directions. Dopaminergic responses were stronger around predictably rewarded correct actions whereas noradrenergic responses were greater around predictably unrewarded correct actions. The complementary response profiles related to the monkeys operant actions suggest that DA neurons might relate to the value of the current action whereas the noradrenergic neurons relate to the psychological cost of that action.

## Introduction

Dopamine (DA) and noradrenaline (NA), two important and closely related modulatory neurotransmitters, are critical for normal motivated behavior. Decreasing either transmitter severely curtails normal exploratory behavior (Slovin et al., 1999; Pessiglione et al., 2006; McGaughy et al., 2008; Sara, 2009; Flagel et al., 2011). The neurons releasing these agents seem to be activated by “salient” stimuli, and the strength of activation seems related to the values of stimuli used for predicting future behavior (Bouret and Sara, 2005; Ravel and Richmond, 2006; Redgrave and Gurney, 2006; Berridge, 2007; Ventura et al., 2007; Bouret and Richmond, 2009; Matsumoto and Hikosaka, 2009; Nomoto et al., 2010; Schultz, 2010). However, DA and NA appear to be related to different functions, with DA being related to assessment of rewards and NA being related to arousal and/or attention. This suggests that their roles in motivated behavior are different in that they appear to reflect different influences of reward on behavior (Robbins and Roberts, 2007; Doya, 2008; Sara, 2009).

Rewards are defined by their appetitive and reinforcing influences on behavior. In addition, they are energizing, in that they enhance arousal and attention. These roles are related but not completely overlapping. We hypothesized that dopaminergic neurons are more sensitive to the incentive value of reward information, its ability to enhance behavior as a function of reward value, whereas noradrenergic neurons are more sensitive to the arousing aspects of reward information, its ability to enhance behavior irrespectively of the value. To examine the hypothesis we extended the analyses of previously presented data. Here, we have compared the activity of noradrenergic neurons from the locus coeruleus (LC) and dopaminergic neurons from the substantia nigra pars compacta (SNc) in different monkeys performing the same reward schedule task (Ravel and Richmond, 2006; Bouret and Richmond, 2009). In this task monkeys behave so that the error rates are directly related to progress through the schedules (Bowman et al., 1996). Because of the relation between the error rates and the schedules we take the error rates to be a direct reflection of the monkey's motivation in the schedules (La Camera and Richmond, 2008). Monkeys master this task quickly (usually within 1–3 testing sessions). Mastery is shown in that the monkeys only make errors when they are not motivated enough to perform the trial. We also measure an appetitive Pavlovian response (lipping). This lipping appears to reflect the subjective value of salient task events such as cue onset or bar release (Bouret and Richmond, 2009, 2010).

The results show that the times at which neuronal responses of SNc and LC neurons occur within this task are similar, in that they occur around the salient events that evoke lipping. However, the modulations of these responses as a function of motivational levels appear to reflect the predicted outcome value for the dopaminergic neurons and the predicted cost to obtain the reward for the noradrenergic neurons. Thus, although these two neuromodulatory systems respond to “salient stimuli,” their influences on target neurons will also be substantially different.

## Materials and methods

The experiments in which the present data were collected were presented in specific articles for the activity in the SNc (Ravel and Richmond, 2006) and the LC (Bouret and Richmond, 2009). Thus, further technical details on the task and the recording methods are available in the corresponding articles.

### Animals

Four adult male rhesus monkeys (*Macaca mulatta*) were used for these experiments, two for each of the structures. The experimental procedures followed the NIH Guide for the Care and Use of Laboratory Animals, and were approved by the NIMH Animal Care and Use Committee.

### Behavior

**Figure 1:**
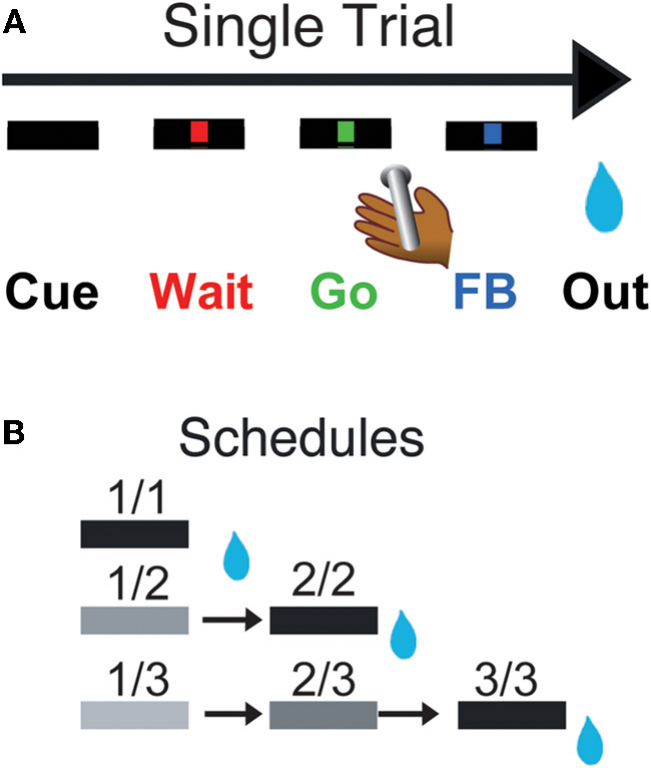
**Reward schedule task.** Each individual trial **(A)** begins if the monkeys touch the bar. A cue (rectangle) appears on the monitor in front of them, followed by a red point (wait signal) after 500 ms. When the point turns green (Go signal, within 500–1500 ms), the monkey must release the bar within 800 ms to complete the trial correctly, in which case the point turns blue (feedback signal). The outcome (juice reward, or not, as a function of the schedule state) occurs 250–550 ms later. In the reward schedule task **(B)**, monkeys must complete either 1, 2, or 3 correct trials to obtain the reward. Once a schedule is completed, another schedule is selected randomly out of the three possible lengths (1, 2, or 3 trials). The progression through the schedules is indicated by the brightness of the cue. All rewarded trials are indicated by a black cue. The fractions indicate the six schedule states, characterized by the trial number (numerator) and the schedule length (denominator). Monkeys must perform a trial correctly to progress through the schedules. If an error is made, monkeys must repeat the erroneous trial but are not require to return to the beginning of the schedule.

Each monkey squatted in a primate chair positioned in front of a monitor on which visual stimuli were displayed. A touch sensitive bar was mounted on the chair at the level of the monkey's hands. Liquid rewards were delivered from a tube positioned between the monkey's lips but away from the teeth. With this placement of the reward tube the monkeys did not need to protrude their tongue to receive rewards. For the experiments where LC neurons were recorded (monkeys D & K), the tube was equipped with a force transducer to monitor the movement of the lips (referred to as “lipping,” as opposed to licking which we reserve for the situation in which tongue protrusion is needed). In the Reward Schedule task, the monkey was required to perform randomly chosen schedules of 1, 2, or 3 color discrimination trials to earn a reward (Figure 1B).

Every trial began when the monkey touched a bar on the chair. A cue appeared; 500 ms later a red instruction target appeared in the center of a monitor 50 cm in front of the monkeys. After another 500–1500 ms, the target turned green. If the monkey released the bar before the green target disappeared 1 s later, the trial was deemed correct, the target turned blue, and during training a liquid reward was delivered. Releasing the bar at any other time was counted as an error, which resulted in an aborted trial. Once monkeys were proficient at that simple task (>80% correct trials), we introduced the reward schedules with their corresponding cues. Cues (horizontal bars of different brightness) appeared at the beginning of each trial and indicated the progression through the schedules (Figure 1B). Cues changed brightness as the schedule progressed, becoming darker as the rewarded trial approached. Schedules of 1, 2, or 3 trials alternated randomly. The monkeys had to complete all trials of a schedule to obtain the reward and before a new schedule was chosen, but errors could be interleaved. After an error, the current trial was restarted that is, the monkeys did not have to return to the beginning of a schedule when they made an error, they only had to complete the current trial. The schedule states will be referred as *i*/*j*, with *i* being the current trial and j the current schedule.

### Electrophysiology

Single unit recording using vertically movable single tungsten electrodes was carried out using conventional techniques. Precise description of the recording procedures can be found in the articles where SNc and LC data used here were originally reported (Ravel and Richmond, 2006; Bouret and Richmond, 2009). Only neurons for which at least 20 correct trials per condition were completed were used in the present analysis, and only data from correct trials was analyzed.

### Data analysis

All data analyses were performed in the R statistical computing environment (Team RDC 2004). After initial inspection of the data, we used a “sliding window” procedure to extract the dynamics of the changes in firing rate in the SNc and LC neurons. We used these data to define six epochs of a trial where neuronal activity was studied further.

#### Sliding window analysis

For each neuron, we counted spikes in a 200 ms test window that was moved in 25 ms increments around the onset of the cue (from −600 to +1200 ms) and around the bar release (from −800 to +1000 ms). Spike counts around each event were standardized so that data from different neurons would be on a common scaling by rescaling them to *z*-scores.

### Response latency

We used the Chi-squared-based procedure described previously to calculate the latency of neuronal activations after cue onset (from 0 to 700 ms) or around bar release (from −400 to 300 ms). Activity in a 100 ms sliding window was compared to background activity from a 500 ms period just before trial start (Ravel and Richmond, 2006; Bouret and Richmond, 2009). A multiple comparisons adjustment was made using the Benjamini and Hochberg fdr procedure (function p.adjust in *R*). For a given event (cue or bar release), responding neurons were defined as those displaying a significant response latency.

#### Response modulation

This analysis was conducted for 6 epochs in each trial. These were “pre-cue” from 400 ms before the cue to cue onset; “cue” from 0 to 500 ms after cue onset; “wait” from 0 to 500 ms after the wait signal; “go” from 250 to 0 ms before the bar release; “blue” from 0 to 250 after the blue point; and “outcome” from 10 to 260 ms after trial outcome. From inspection it appeared that the modulation of firing across schedule states fell into four categories: those where the activity (1) was indistinguishable across all schedule states, (2) differed between first and non-first trials in the schedule (“First” modulation), (3) differed between rewarded and unrewarded trials (“Reward” modulation), and (4) showed idiosyncratic response patterns across the six schedule states (“State” modulation). For each neuron, activity in each epoch was tested with a 6-level One-Way ANOVA, where the six levels were the six schedule states. To evaluate whether this cue selectivity arose because there was first-non first or reward-no reward selectivity, the responses were subjected to two additional ANOVAs: a 2-level ANOVA where the levels coded whether the schedule state was a first trial or not, and a 2-level ANOVA where the levels coded whether the schedule state was rewarded or not. If the first-non-first test was significant, the One-way 2-level ANOVA model was tested against the 6-level ANOVA model. This latter procedure determines whether the extra degrees of freedom in the 6-level ANOVA are justified. If the difference was not significant, the simpler model, i.e., the 2-level ANOVA, was preferred and the firing modulation was classified as First. The same comparison between the 2-levels reward-no reward and the 6-levels ANOVA was used to identify Reward modulation.

#### Timing of the firing modulation

We quantified the dynamics of the firing modulation by measuring two variables: modulation latency and peak latency, using a sliding window procedure. For the cue, we focused on a 1 s period starting 400 ms before cue onset. For the bar release, we focused on a 1 s period centered on the bar release. For each neuron, we used a sliding window procedure similar to the one described above for the rate analysis (200 ms test windows moved in 25 ms increments). Here, at each step, we computed 2 One-Way ANOVAs: one for the First effect (2 levels, first and non-first) and one for the Reward effect (2 levels, rewarded and non-rewarded). For a given effect (First or Reward), the modulation latency was defined as the first of three successive windows with a significant effect, after correction for multiple comparisons (FDR procedure). The peak latency of a given effect was defined as the window in which the effect was maximal, whether it was significant or not.

## Results

### Reward schedules and motivation

Monkeys were taught a Reward Schedule task, in which they performed schedules of one, two, or three operant sequential color discrimination trials correctly to obtain a reward (Figure 1). Each trial has the same operant demands: touch a bar, watch a red target spot, when the target turns green release the bar. If the bar is released before the green disappears (a correct response), the target turns blue (feedback signal), and during training a drop is delivered for every correct trial. During the Reward Schedule task, if the trial is the last in a schedule, a drop of liquid reward is delivered. If the trial is not the last the monkey progresses to the next trial in the schedule. Progression through the schedules is indicated by the brightness of the cue (rectangle) presented at the beginning of each trial. Trials ending in errors (releasing before green appears or after it disappears) are repeated, without resetting the schedule. A new schedule is chosen randomly after a reward is earned. We measured two types of behavior: an operant response (bar release) and a Pavlovian response (lipping). The comparison between these two types of behavior allows us to estimate the relation between the value of different task events (using lipping) and operant performance across conditions. The relative sensitivity of neuronal activity in SNc and LC neurons to these different aspects of behavior will also be characterized.

**Figure 2:**
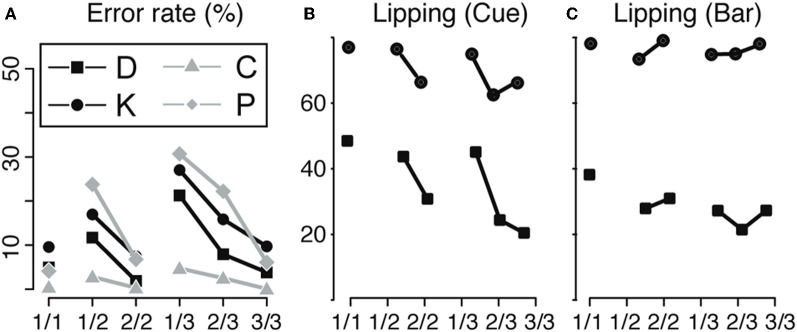
**Behavior. (A)** Bar release. Error rate for the operant response (bar release) across the six schedule states, indicated by fractions, for the four animals (D, K, C, and P). The error rate is minimal in rewarded trials (1/1, 2/2, and 3/3), indicating that monkeys knew the task and were motivated enough to work when a reward was immediately available upon trial completion. In non-rewarded trials, the error rate increased with the distance to the reward, indicating a decrease in motivation to perform the operant response when more effort/time was required to obtain the reward. **(B,C)** Lipping behavior. Percentage of trials with a lipping response at the cue **(B)** and the bar release **(C)** across the six schedule states (indicated by fractions), for monkeys D and K. Lipping is a Pavlovian appetitive response that we use to assess the value of an event (cue onset or bar release). At the cue **(B)**, lipping was more prominent in the first trials of a schedule (1/1, 1/2, and 1/3), whether or not the trial was immediately rewarded. The first cue of a schedule signals that a schedule begins and that a reward will be obtained upon its completion. Subsequent cues carry redundant, less valuable information. At the bar release **(C)**, lipping was greater in rewarded trials (1/1, 2/2, and 3/3) than in unrewarded trials, in line with the bar release performance shown in **A**. Replotted from Bouret and Richmond (2009) and Ravel and Richmond (2006).

Even though each trial has the same operant demand, the monkeys make different numbers of errors in different trials of the schedules (Figure 2A). Because the monkeys must repeat error trials, it would seem that there is no incentive to make an error, so the most parsimonious interpretation is that monkeys make more errors when they are not motivated enough to perform well (Bowman et al., 1996; Bouret and Richmond, 2009). In all four monkeys, error rates were highest in unrewarded trials that were furthest from reward, decreased as the monkeys progressed through the schedules and were smallest in rewarded trials (significant effect of schedule states on error rate, ANOVA, *p* < 0.05 for all monkeys). This indicates that the predicted outcome value was discounted by the time and/or effort required to obtain the reward.

We used the frequency of lipping in correctly performed trials to estimate the subjective value of these two task events (Bouret and Richmond, 2010). At the cue (Figure 2B), the proportion of correctly performed trials with lipping responses was greater for correctly performed first trials of a schedule, whether they were to be rewarded or not, than for correctly performed non-first trials (monkey D: 46% vs. 25% χ^2^(1) = 179, *p* < 1 × 10^−10^; Monkey K: 76% vs. 65%, χ^2^(1) = 70, *p* < 1 × 10^−10^). The amount of lipping was the same in the 1-trial schedules as in the first of the two- or three-trial schedules. Thus, the value of the trial is related to it being first, not whether it is rewarded, presumably because first cues are the most informative in that they predict both that a reward will be available (upon schedule completion) and when (how many trials) (Bouret and Richmond, 2009), that is, the first cues remove all uncertainty about the current schedule. At bar release (Figure 2C), the proportion of lipping responses was higher in rewarded than in correctly performed unrewarded trials [D: 32% vs. 25% (χ^2^(1) = 22, *p* = 5 × 10^−6^); K: 78% vs. 74% (χ^2^(1) = 12, *p* = 0.0006)]. We interpret this as indicating that bar release is most valuable when it leads to an immediate reward. Thus, the error rates and lipping responses do not covary, make it seems likely that the operant performance (best operant performance in rewarded trials) is not simply a reflection of a Pavlovian influence of the cue (strongest lipping in first trials). If the operant response had been due to the Pavlovian influences, we would have found that Pavlovian responses and the operant performance would have covaried, that they did not. Thus, it appears that evaluation of the cue provides two separate types of information. One type reflects the predicted cost (how many trials left in a schedule) and the other reflects the benefit of performing the current trial (is there a reward at stake). The overall operant performance arises from combining these pieces of information. On the other hand, the Pavlovian response seem to reflect that a reward will be available (at the completion of a schedule), irrespectively of the predicted cost, the number of trials necessary to obtain it.

### SNc and LC neurons respond to salient events

**Figure 3:**
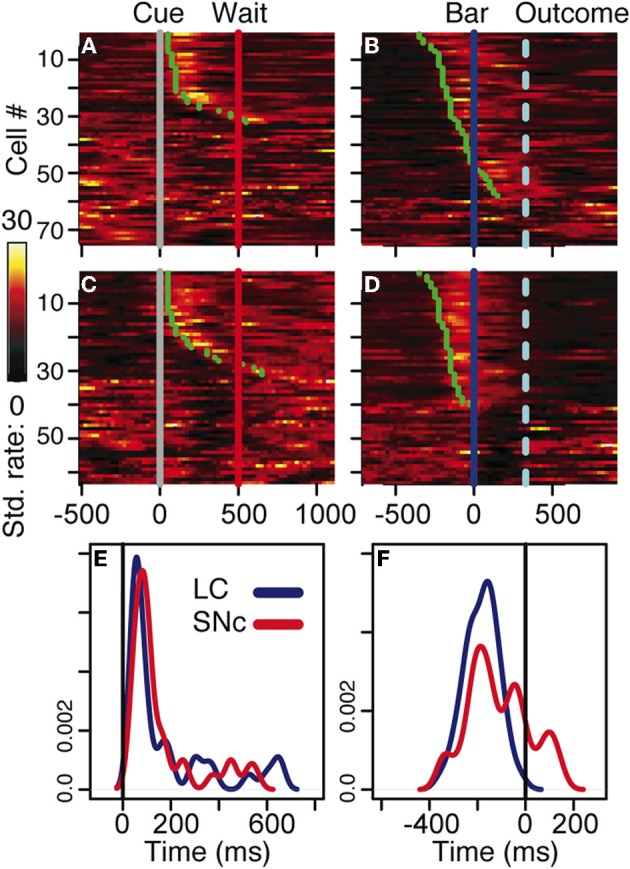
**Neuronal activity. (A–D)** firing rate of the population of SNc **(A–B)** and LC **(C–D)** units at the cue **(A,C)** and the bar release **(B,D)**. In all four plots, each line is the color-coded standardized (*z*-scored) firing rate of a single neuron around the event of interest. An additional line indicates the time of the wait signal relative to cue onset, and the average time of the outcome relative to the bar release. Neurons are sorted by increasing latency of the response (green dot). At the cue, about half of the neurons in SNc **(A)** and LC **(C)** display a transient activation at cue onset, with a similar timing between the two populations. A comparison of the distribution of SNc and LC response latencies at cue onset **(E)** confirms that the timing of the responses of these two populations was undistinguishable. At the bar release, both SNc **(B)** and LC **(D)** neurons display a transient activation right before the bar release. In the LC, the activation of all responding neurons started before the bar release whereas in the SNc, neurons started to respond both before and after the bar release. This was confirmed when we plotted the distribution of response latencies around bar release for these two populations **(F)**.

**Figure 4:**
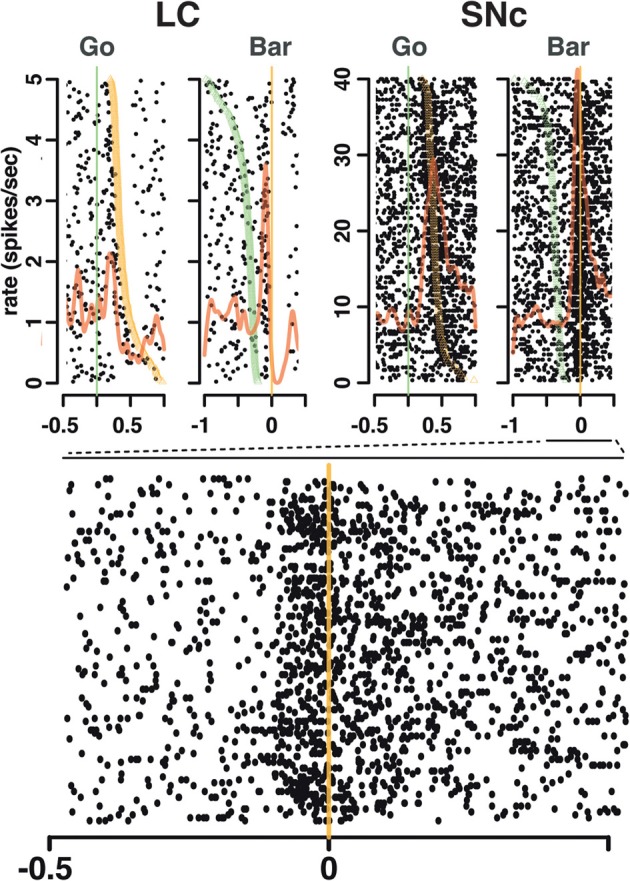
**Spike timing around the go signal and the go response.** Neuronal activity of a representative SNc neuron (left) and a representative LC neuron (right). For each neuron, raster and spike density were generated with spikes aligned either on the onset of the go signal (green line, left) or the ensuing bar release (orange line, right). Trials are sorted by decreasing reaction time, with orange triangles representing the time bar release in each trial on the go signal-aligned activity and green triangles representing the go signal on the bar release aligned activity. For both the SNc and the LC, spikes fired between the go signal and the bar release were more closely related to the triggering of the action than to the onset of the stimulus.

We analyzed all recorded neurons for which we had at least 20 correct trials per schedule state. The dataset comprises the activity of 75 dopaminergic SNc neurons [from two monkeys, C and P; (Ravel and Richmond, 2006)] and 63 noradrenergic LC neurons [from two other monkeys, D and K; (Bouret and Richmond, 2009)]. The behavior of the two pairs of monkeys used in these experiments were undistinguishable. The average firing rate (over the whole recording session, including excitatory responses) of SNc neurons (7 ± 0.4 spk/s) was significantly higher than that of LC neurons (2 ± 0.2 spk/s, *t*_(1)_ = 11.7; *p* < 10^−8^). At first sight, the response patterns of SNc and LC neurons in this task appear to be similar: in both areas, neurons display a transient activation following the cue and around bar release (Figure 3), with a stronger activation around the bar release (SNc: 8.2 ± 0.8 spk/s, LC: 3.5 ± 0.3 spk/s) than after the cue onset (SNc: 6.9 ± 0.4 spk/s, LC: 2.1 ± 0.2 spk/s). We examined the spiking activity around bar release in correctly performed trials to determine whether the activation was more closely related to the go signal or to the action itself. Representative examples are shown on Figure 4. For each neuron, we measured the times of spikes fired between the onset of the go signal and the bar release and compared their distribution aligned on each of these events. Since the total number of spikes was the same in the two distributions, a higher peak rate indicates better alignment across trials. For both SNc and LC neurons, the peak firing rate was significantly higher when spikes were aligned to the bar release rather than to the go signal (paired *t*-test on the peak value of the 2 distributions, *p* < 0.05 for each of the 2 areas); that is, spikes were better aligned to the bar release than the go signal. For LC responses, this is in line with previous descriptions of LC activity in rats and monkeys (Bouret and Sara, 2004; Rajkowski et al., 2004) and here it is true for dopaminergic SNc neurons, also. This does not mean that the activation of LC and SNc neurons is simply related to the movement itself. As has been reported by others for LC neurons, neither the LC nor the SNc neurons were activated when bar release occurred outside of this context, such as between trials (Bouret and Sara, 2004; Rajkowski et al., 2004; Bouret and Richmond, 2009). At the cue, 32 (43%) SNc neurons and 31 (49%) LC neurons showed a significant increase in firing rate, with mean latencies of 100 (IQR = 75–175) and 75 ms (IQR = 50–250), respectively (Figures 3A,C, and E). Both the proportion of responding neurons and the response latencies were indistinguishable between the 2 regions (χ^2^ and Wilcoxon, *p* > 0.05). These latencies are within the same range as previous reports (112 and 118 ms, for SNc and LC, respectively), even though the analysis differs slightly (Ravel and Richmond, 2006; Bouret and Richmond, 2009). The latencies reported here probably appear shorter because the ones here come from the data grouped across states and are likely to represent the shortest latency across states, i.e., they represent the extreme statistic of the minimum across schedule states. Nonetheless, the latencies from the two analyses are statistically indistinguishable. We previously showed that response latencies were equivalent across schedule states for each of these two populations of neurons. Based on the similarity in activation timing in SNc and LC neurons it is possible that this represents a common or overlapping drive. At the bar release (Figures 3B and D), 58 (77%) SNc neurons and 40 (63%) LC neurons showed a significant response (no difference in proportion, χ^2^, *p* > 0.05). The distribution of LC response timing was narrower than that of SNc responses (Kolmogorov–Smirnov test on response latencies between the 2 areas: *p* = 0.005), with all 40 LC neurons starting to respond before the bar release. Although a substantial number of the SNc neurons also start responding before bar release, a significant number of SNc neurons start responding only after the bar release (Figure 3F). Thus, both SNc and LC neurons are activated around the bar release but the timing of that activation is more consistent, and, hence, on average earlier in the LC (where all responses occur before the bar release) than in the SNc (where neuronal responses occurred both before and after bar release).

In the LC, neurons that were not excited following the cue (Figure 3C) or before bar release (Figure 3D) displayed a transient decrease in firing rate after the peak of the activation of the neurons showing a response. This seems like the effect described in earlier work investigating an alpha-2 dependent auto-inhibition of noradrenergic LC neurons (Aghajanian et al., 1977). The mechanism underlying this decrease in firing in LC neurons lacking an excitatory response may also account, at least in part, for the abrupt termination of the excitatory response in responding neurons.

Overall, both SNc and LC neurons are activated at the cue and at the bar release, two salient events in the task. The proportion of responding neurons was about the same in the two areas. At the cue, the latency was the same, whereas at the bar release, the latency of LC responses was more homogenous than for SNc responses.

### Modulation of SNc and LC responses related to event value

We measured neuronal activity in each of six epochs of a trial: “pre-cue” (from 400 ms before cue to cue onset); “cue” (from 0 to 500 ms after cue onset); “wait” (from 0 to 500 ms after the wait signal); “go” (from 250 to 0 ms before the bar release); “blue” (from 0 to 250 after the blue point); and “outcome” (from 10 to 260 ms after trial outcome). For each epoch we first used a 6-level One-Way ANOVA to determine whether the activity was related to the schedule (one-level for each schedule state in the task). Neurons for which responses were indistinguishable across the six schedule states were not considered further. From inspection, it appeared that the modulation of firing across the six schedule states fell into three categories: (1) First modulation, where activity differed mostly between first and non-first trials, (2) Reward modulation, where activity differed mostly between rewarded and unrewarded trials, (3) State modulation, where activity showed an idiosyncratic pattern across the six states. To explore this, we carried out model comparisons. For each neuron the 6-level ANOVA was compared with each of two 2-level ANOVAS (one where the 2-levels were first/non-first and one where the two levels reward/no-reward). If the 6-level ANOVA was not significantly better at explaining the data (compared by ANOVA using the anova function in R), the neuron was assigned by the 2-level result (First or Reward) that accounted for the most variance, otherwise it was left as a State modulation.

**Figure 5:**
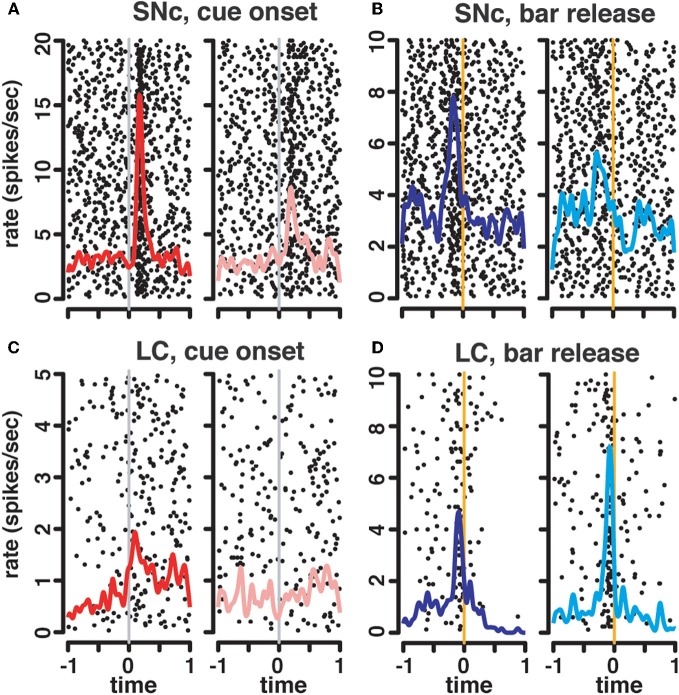
**State-dependent modulation of neuronal responses.** Representative raster plots and spike density functions for 2 representative SNc units **(A–B)** and 2 LC units **(C–D)**. For responses at the cue, we plotted activity recorded in first trials (1/1, 1/2, and 1/3) in red (left) separately from activity recorded in non-first trials (2/2, 2/3, 3/3; pink, right). For this SNc unit **(A)**, the activation at the cue was greater in first compared to non-first trials. The LC unit **(C)** only responded to cues signaling the first trial of a schedule, but not subsequent ones. At the bar release, trials were sorted according to whether they were rewarded (dark blue, left), or not (light blue, right). For the SNc unit **(B)**, there was a greater activation before rewarded bar releases compared to unrewarded ones. On the contrary, in the LC **(D)**, the activation before the bar release was greater in non-rewarded trials.

**Figure 6:**
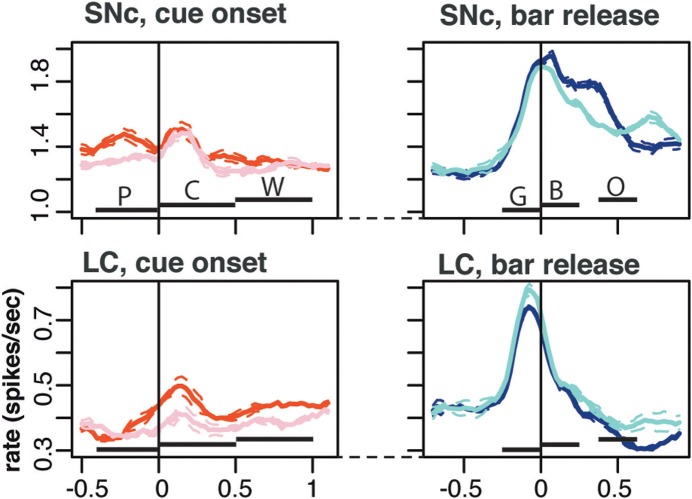
**Population activity.** Average population firing for the entire population of SNc (top) and LC neurons (bottom). All recorded neurons were included, whether they showed task related activity or not. We plotted the average activity around two events of a trial: cue onset (left) and correct bar release (right). Around cue onset (left), we separated 1st from non-first trials in a schedule. The horizontal bars indicate the standard epoch used for the analysis, “pre-cue” (P, from 400 ms before the cue to cue onset); “cue” (C, from 0 to 500 ms after cue onset); “wait” (W, from 0 to 500 ms after the wait signal); “go” (G, from 250 to 0 ms before the bar release); “blue” (B, from 0 to 250 after the blue point); and “outcome” (O, from 10 to 260 ms after trial outcome). The first three epochs are time-locked to each other, then there is a variable period at the end of the wait, followed by the go, blue, and outcome that are also almost completely time-locked (there is a 250 ms of jitter in outcome timing). Around bar release (right), we separated rewarded and unrewarded trials. Each trace was generated by averaging firing rates across all recorded neurons in the corresponding subset of trials. The broken lines represent the error (SEM) envelop around the mean. The baseline rates were so similar in general that no normalization of firing rates across neurons was used. On average, the firing of both SNc neurons was greater in first trials around cue onset. Around bar release, the average firing was greater in rewarded trials for SNc neurons but greater in unrewarded trials for the population of LC neurons.

**Figure 7:**
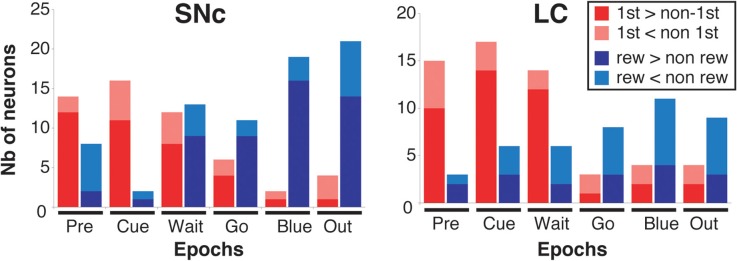
**Response modulation across epochs of a trial.** Number of neurons showing a significant modulation of their firing between first and non-first (First modulation) or between rewarded and unrewarded trials (Reward modulation) in the SNc **(left)** and the LC **(right)**, across the six epochs of a trial (see text and Figure 6 for a description of these epochs). The analysis was conducted on the entire population of recorded neurons (SNc: *n* = 75, LC: *n* = 63), without any selection based on their activity in the task. Each bar indicates the total number of neurons showing a significant modulation. Within each epoch, for neurons showing a significant modulation between rewarded and unrewarded trials, we separated neurons for which the firing rate was greater in rewarded trials (dark red) from neurons for which the firing rate was greater in unrewarded trials (light red). Within each epoch, for neurons showing a significant modulation between first and non-first trials, we separated neurons for which the firing rate was greater in first trials (dark blue) from neurons for which the firing rate was greater in unrewarded trials (light blue).

**Figure 8:**
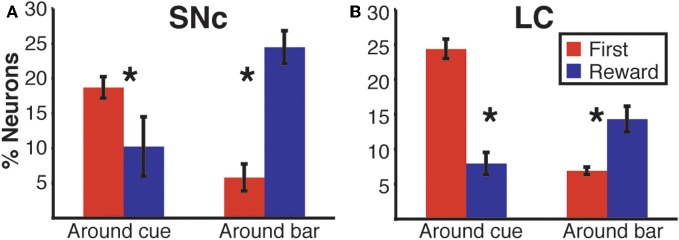
**Response modulation across epochs of a trial.** *Average* percentage of neurons displaying a modulation of their firing between first and non-first (First modulation) or between rewarded and unrewarded trials (Reward modulation) in the SNc **(A)** and the LC **(B)**. Based on the results in Figure 7, where the first-non-first and reward-noreward groups showed the same patterns, we grouped the data from the first three epochs and the last three epochs. Each bar is the mean and SEM of three measures taken at the beginning (1 point per epoch, before cue, after cue and after the wait signal) or the end of a trial (before bar release, after bar release, after the outcome). In both populations, the modulation of neuronal activity at the beginning of a trial (around cue onset) mostly depends upon whether the trial was the first of a schedule, or not. At the end of a trial, around bar release, neuronal activity gets predominantly modulated according to whether trials are rewarded, or not. Stars: significant difference (*p* < 0.05).

In both regions, it was clear from inspection that the nature of the modulation changed markedly over the course of a trial. Early in the trial, around cue onset, neuronal activity in both regions was mostly modulated as a function of whether the trial was the first of a schedule, or not (First modulation, Figures 5A and C). Later on during the trial, around bar release, neuronal activity was mostly modulated as a function of whether the trial was rewarded or not (Reward modulation, Figures 5B and D). This pattern was seen in the population activity across all of the neurons (Figure 6). We compared the proportions of neurons displaying significant First and Reward modulations for each of the six measurement epochs using a Two-Way ANOVA (Figures 7 and 8). One factor was “type of modulation,” First and Reward, and the other factor was “measurement epochs” grouped into 2 levels, Beginning (epochs 1–3) and End (epochs 4–6). For both SNc and LC, the interaction between the 2 factors was significant (SNc: *F*_(1, 8)_ = 17, *p* = 0.003; LC: *F*_(1, 8)_ = 96, *p* = 1 × 10^−5^). We chose the 2 levels Beginning (first 3 epochs) and End (last 3 epochs) because within each level the 3 epochs are continuous, non-overlapping, and the patterns of response, whether first vs. non-first, and reward vs. no reward, were similar across the first 3 and the last 3. Based on this finding, we focused on the First modulation around cue onset, at the beginning of a trial, and on the Reward modulation around bar release, at the end of a trial, for the remainder of the analysis.

We used a sliding window procedure to describe the dynamics of the firing modulation (see Materials and Methods). We measured the modulation latency (time at which the firing modulation across schedule states started in individual neurons) and the peak latency (time at which the firing modulation was maximal, whether it was significant or not). At the cue, the average First modulation latencies and the peak latencies were indistinguishable across the 2 regions (modulation latencies: −158 ± 55 ms and −150 ± 55 ms in the SNc and the LC, respectively, *t*_(1)_ = 0.1; *p* = 0.9; peak latencies: 115 ± 32 ms and 69 ± 35 ms in the SNc and the LC, respectively; *t*_(1)_ = 1; *p* = 0.3). In both structures, the First modulation clearly emerges before cue onset, but the cue is not needed since a first trial is marked in that it is a trial following a reward (Bouret and Richmond, 2009). At bar release, the average Reward modulation latencies were also indistinguishable between the 2 regions (−41 ± 44 ms and −31 ± 100 ms in the SNc and the LC, respectively, *t*_(1)_ = 0.1; *p* = 0.9). However, the peak latency was significantly shorter in the LC (−23 ± 35 ms) than in the SNc (66 ± 29 ms, *t*_(1)_ = 2; *p* = 0.05), indicating that the modulation of firing according to whether there was a reward or not rose more quickly in the LC (before bar release) than in the SNc (after bar release).

### Firing modulation differs between SNc and LC

**Figure 9:**
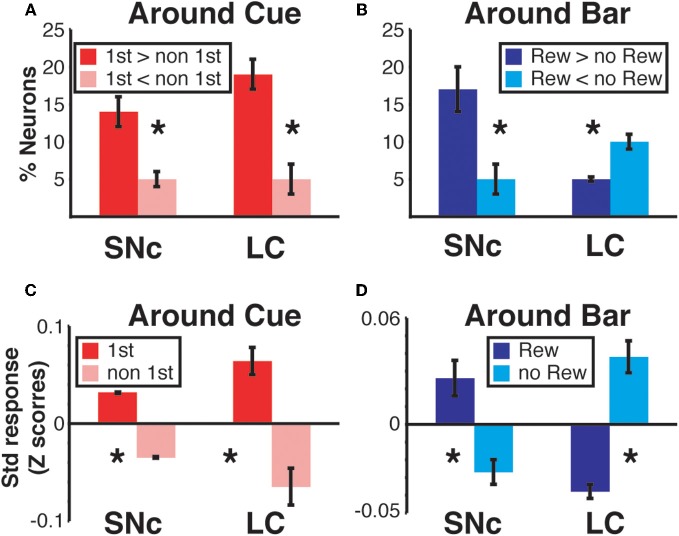
**Region specific response modulation. (A)** Percentages of SNc and LC neurons displaying a First effect at the beginning of a trial (around cue onset), broken down into neurons for which the response was greater in first trials or greater in non-first trials. In both regions, a majority of neurons were more active in first compared to non-first trials. **(B)** Percentages of SNc and LC neurons displaying a Reward effect at the end of a trial (around bar release), broken down into neurons for which the response was greater in rewarded trials or greater in unrewarded trials. In the SNc, most of the neurons displaying a reward effect were more active in rewarded trials. In the LC, a majority of the neurons showing a Reward effect were more active in unrewarded trials. **(C)** Standardized population responses of SNc and LC neurons in first and non-first trials at the beginning of a trial. For both regions, the firing rate of the whole population was clearly greater around cues indicating the first trial of a schedule, compared to subsequent ones. **(D)** Standardized population responses of SNc and LC neurons in rewarded and unrewarded trials at the end of a trial. In the SNc, the population firing around the bar release was greater in rewarded trials. On the contrary, in the LC, activity around the bar release was greater in unrewarded trials compared to rewarded trials. Each bar is the mean ± SEM of three epochs across three schedule states. Stars: significant difference (*p* < 0.05).

We sorted neurons showing First (or Reward) modulation according to whether their firing rate was higher or lower in first vs non-first trials (or reward vs. unrewarded reward trials, respectively) (Figures 7 and 9). At the beginning of a trial, in both regions, most of the neurons displaying a significant First modulation had stronger firing in first than in non-first trials (Figure 9A). At the end of a trial, however, the two areas differed (Figure 9B). In the SNc, a majority of modulated neurons fired more strongly in rewarded than unrewarded trials, whereas in the LC, the firing was weaker in rewarded trials for the majority of neurons. In other words, around bar release, the firing of LC neurons was greater in unrewarded trials whereas the firing of SNc neurons was greater in rewarded trials.

We constructed a population activity graph by calculating the mean standardized (*z*-score) firing of all neurons, whether they were responding or not, in each of the six epochs. At the beginning of a trial (epochs 1–3), the average standardized firing was greater in first than in non-first trials in both structures (Figure 9C; Two-Way ANOVA), no effect of “region,” SNc vs. LC, *F*_(1)_ = 0.08; *p* = 0.9; significant effect of “modulation” (first vs. non-first, *F*_(1)_ = 47; *p* = 9.5 × 10^−8^). At the end of trials (epochs 4–6) the population activity was larger in rewarded trials for SNc neurons and larger in unrewarded trials for LC neurons (Figure 9D; Two-Way ANOVA), no effect of “region,” SNc vs. LC, *F*_(1)_ = 0.07; *p* = 0.9; no effect of “modulation” (rewarded vs. non-rewarded, *F*_(1)_ = 1.7; *p* = 0.2; significant interaction between the 2 factors: *F*_(1, 32)_ = 53, *p* = 2.6 × 10^−8^). Thus, the pattern seen with the individual neurons showing a significant effect (Figures 9A,B) was present when all the neurons in a given region were considered together, without any kind of selection based on their activity (Figures 9C,D).

## Discussion

In comparing the responses of SNc and LC neurons in monkeys during reward schedules, we find that the responses of SNc and LC neurons have strong similarities, as might be expected given their likely evolutionary history. We identify two salient events, cue appearance and go signal/bar release, by their proximity to lipping, an appetitive reflex which has characteristics of a Pavlovian response (Bouret and Richmond, 2009, 2010). Other Pavlovian responses including autonomic responses have also been reported at the time of reward predicting cues and at the time of goal-directed responses (Collet et al., 1999; Amiez et al., 2003; Pavlov, 2003). Taken altogether, the evidence suggests that the activation of both SNc and LC neurons might be related to the reflex responses evoked by behaviorally significant stimuli. Because these neuromodulatory nuclei share a strong interconnection with subcortical structures controlling autonomic functions and simple reflex behaviors (Aston-Jones et al., 1991; Bouret et al., 2003; Phillips et al., 2003; Dommett et al., 2005; Okada et al., 2009; Hazy et al., 2010), one possiblity raised by the results here is that the DA and NA systems are activated by common inputs from these subcortical structures controlling reflex responses to salient events. In the case of the NA system, there is ample evidence that LC activity is strongly related to arousal and autonomic responses, and the present data indicate that DA neurons might be driven, at least to some extent, by similar processes (Aston-Jones et al., 1991; Berridge and Waterhouse, 2003).

Our analysis of the neuronal responses for the go signal/bar release shows that they are better aligned to the bar release than to the go signal so as a shorthand we describe them as bar release related. However, the responses are not directly driven by the bar release itself since the neurons did not respond to bar releases between trials, that is, they did not respond outside of the context of the task. Furthermore, the spread of the timing of the discharges across the population of SNc neurons, mostly before but sometimes after, the motor response, makes it unlikely that the SNc responses are directly related to the motor response. In general the responses of SNC neurons are interpreted in light of sensory events. In that light, the responses could be, for example, related to some unmeasured sensory signal that is better time-locked to bar release than to the go signal, perhaps a visual signal arising from a stereotyped eye movement better synchronized to the bar release than to the imperative go signal, or to the stereotyped eye movement itself. In a similar fashion, the activation observed shortly after cue onset could be related to an unmeasured eye movement and/or a corresponding change in sensory stimulation. Both at the cue and between the go signal and the bar release, neuronal activation could be related to changes in internal state or cognitive operations that we can not measure. It is even possible that there is a corollary such as been proposed for eye movements by Redgrave and Gurney (2006). Given all of these caveats, inferring the origin of the signal is difficult. What we can say is that both SNc and LC neurons are activated shortly after the onset of the cue and around the time of the bar release, with similar timings between the 2 structures, without passing any judgment regarding the source of the activation.

The modulation of the responses of catecholaminergic neurons across schedule states follows the same pattern as the modulation of lipping responses, in that their firing is mostly affected by the factor “first/non-first” at the cue and by the factor “reward/no-reward” around the bar release. Thus, the activation of these neurons is not only related to the timing of these salient events, but also to their relative value, as measured by the proportion of lipping responses across schedule states (Bouret and Richmond, 2010). We speculate that catecholaminergic neurons receive value information from ventral prefrontal cortices, which are involved in value processing (Chiba et al., 2001; Padoa-Schioppa and Assad, 2006; Lebreton et al., 2009; Bouret and Richmond, 2010). A similar firing pattern has been observed in ventral prefrontal activity in several versions of this task, and value assessment is impaired after bilateral lesions of these areas (Simmons and Richmond, 2008; Bouret and Richmond, 2010; Simmons et al., 2010).

At the bar release, even though in formal informational terms the responses of both sets of neurons are similar in that there is a response difference between rewarded and non-rewarded trials, the manner through which this information is realized is inverted in the two systems. In the SNc, the firing rate parallels the lipping behavior in that the responses tend to be stronger in trials when there is a reward forthcoming than in trials when there is no reward forthcoming. This result is consistent with the finding that DA neuronal firing is modulated in relation to value (Schultz et al., 1997; Berridge, 2007; Schultz, 2010; Flagel et al., 2011). However, it does not seem straightforward to relate our data to the idea that the DA responses encode reward prediction error. The prediction error hypothesis states that the firing of DA neurons is related to the error between what is expected and what actually occurs, the difference being an error signal that would drive learning, especially reinforcement learning (Schultz et al., 1997). At the time of the imperative green/bar release event the outcome of the current trial was certain, something the monkeys clearly knew and responded to in that they made almost no errors in rewarded trials. Based on the prediction error hypothesis one might expect, either that the DA neurons would not fire at all because the outcome was just about certain, or that the DA signal would be larger in unrewarded trials because these are trials where what we define to be an error is more likely. We are cautious in calling these “errors,” where error might imply that the monkeys do not know what is expected. In our reward schedule task the monkeys can and apparently do predict the outcomes for every trial from the instant the cue appears. The “errors” probably represent a type of refusal related to frustration when the monkeys know that there is no reward immediately forthcoming. In these most certain of trials, where the outcome depended on the monkey's reaction to the imperative go signal, the population of DA neurons fired in both rewarded and unrewarded trials, with the firing on average stronger in rewarded trials. In addition, although not repeated here, our previous analysis did not reveal any systematic difference in the first cue responses, even though the first cue in a 3-trial schedule predicted reward that was three times as far away in time as a one trial schedule (Ravel and Richmond, 2006). This is in line with recent data showing a relative lack of sensitivity of the DA system to expected cost (Gan et al., 2010). Thus, it seems difficult to apply the reward prediction error hypothesis to our data. Our data do support the interpretation that DA neurons fire and at least in part encode the value associated with salient events (Dommett et al., 2005; Redgrave and Gurney, 2006; Berridge, 2007; Matsumoto and Hikosaka, 2009).

Actions are triggered when motivation crosses some threshold. We propose that the motivation needed to reach this threshold can come from at least two sources. The first is when the value of a reward is great enough by itself to reach the threshold for action. However, there are circumstances when the value of the reward itself may not be enough to trigger the action, for example, in unrewarded trials of our reward schedule task. We propose that LC neurons have the appropriate firing profiles to be responsible for the additional motivation. Because LC neurons fire more strongly at the action in unrewarded trials, we speculate that the activation of LC neurons is related to the fact that there is higher cost to continue the trial, for example, to act respond no reward is immediately forthcoming, whereas in rewarded trials where the motivation being driven by reward is high, the cost is low and therefore the reward value reflected in the dopamine responses is enough to trigger the bar release response. Here the motivational level driven by the reward value (measured using the error rate) increases as monkey's progress through the schedules. In other words, if an action is triggered when a hypothetical value threshold is crossed, the threshold is more likely to be reached in rewarded trials because the motivation coming from the reward value is higher. In this scenario the reward-related motivation in unrewarded trials would be further away from the threshold. What we refer to as cost here would be related to the amount of motivation that must be added to the reward-dependent level to cross the threshold for triggering the action. In this framework, the larger response by LC neurons to first cues compared to non-first cues would occur because first cues require additional information processing, and thereby represent a greater cost (investment), compared to non-first cues, which are confirming what is already known. This model of LC neurons would account for data showing a critical role in functions that could also be described as particularly “effortful” (Berridge and Waterhouse, 2003; Bouret and Sara, 2005; Yu and Dayan, 2005; McGaughy et al., 2008). A similar response pattern was recently described in the CM/PF thalamus, which is thought to play a role in promoting behaviors that are required, even if the expected benefit is relatively low (Minamimoto et al., 2005, 2009). Perhaps the LC and the CM/PF thalamus belong to a common functional ensemble that would play a role complementary to that of the dopaminergic system in motivation. Recent studies indicate that the dopaminergic system is relatively insensitive to the cost/effort aspects of outcome values (Gan et al., 2010; Wanat et al., 2010). In the hypothesis proposed here, the encoding of cost/effort would be reflected in the noradrenergic system. In other words, NA would promote costly behaviors by increasing the likelihood to act when the reward or goal-related motivation is not enough to support the activity needed to pursue a deferred goal, whereas DA would promote behaviors when the immediate expected benefits are high.

Overall, these two populations of catecholaminergic neurons are activated in close proximity to the same behaviorally significant events in our task, in line with the idea that they evolved from a common ancestor. We propose that this ability to respond to salient events, presumably through inputs from relatively primitive brain regions, emerged early during evolution and is similar across vertebrate species, whereas the firing diverged so that SNc neurons track the relative outcome value whereas the firing of LC neurons appears to reflect the cost to continue the current behavioral program. The finer functional properties, which presumably rely on inputs from telencephalic structures, appear less well conserved. It is widely recognized that these two groups of neurons have different connectional patterns, and that the receptors for these catecholamines are distributed widely, but reflect the different connectional patterns. The present data show that the two groups of neurons show different modulation patterns in relation to the same task events, and these patterns can be interpreted in terms of their contributions to ongoing behavior.

### Conflict of interest statement

The authors declare that the research was conducted in the absence of any commercial or financial relationships that could be construed as a potential conflict of interest.

## References

[B1] Aghajanian G. K. Cedarbaum J. M. Wang R. Y. (1977). Evidence for norepinephrine-mediated collateral inhibition of locus coeruleus neurons. Brain Res. 136, 570–577 10.1016/0006-8993(77)90083-X 922502

[B2] Amiez C. Procyk E. Honoré J. Sequeira H. Joseph J.-P. (2003). Reward anticipation, cognition, and electrodermal activity in the conditioned monkey. Exp. Brain Res. 149, 267–275 10.1007/s00221-002-1353-9 12632229

[B3] Aston-Jones G. Shipley M. T. Chouvet G. Ennis M. van Bockstaele E. J. Pieribone V. A. Shiekhattar R. Akaoka H. Drolet G. Astier B. (1991). Afferent regulation of locus coeruleus neurons: anatomy, physiology and pharmacology. Prog. Brain Res. 88, 47–75 1687622 10.1016/s0079-6123(08)63799-1

[B4] Berridge C. W. Waterhouse B. D. (2003). The locus coeruleus-noradrenergic system: modulation of behavioral state and state-dependent cognitive processes. Brain Res. Brain Res. Rev. 42, 33–84 10.1016/S0165-0173(03)00143-7 12668290

[B5] Berridge K. C. (2007). The debate over dopamine's role in reward: the case for incentive salience. Psychopharmacology 191, 391–431 10.1007/s00213-006-0578-x 17072591

[B6] Bouret S. Sara S. J. (2004). Reward expectation, orientation of attention and locus coeruleus-medial frontal cortex interplay during learning. Eur. J. Neurosci. 20, 791–802 10.1111/j.1460-9568.2004.03526.x 15255989

[B7] Bouret S. Sara S. J. (2005). Network reset: a simplified overarching theory of locus coeruleus noradrenaline function. Trends Neurosci. 28, 574–582 10.1016/j.tins.2005.09.002 16165227

[B8] Bouret S. Duvel A. Onat S. Sara S. J. (2003). Phasic activation of locus ceruleus neurons by the central nucleus of the amygdala. J. Neurosci. 23, 3491–3497 12716958 10.1523/JNEUROSCI.23-08-03491.2003 PMC6742334

[B9] Bouret S. Richmond B. J. (2009). Relation of locus coeruleus neurons in monkeys to Pavlovian and operant behaviors. J. Neurophysiol. 101, 898–911 10.1152/jn.91048.2008 19091919 PMC2657074

[B10] Bouret S. Richmond B. J. (2010). Ventromedial and orbital prefrontal neurons differentially encode internally and externally driven motivational values in monkeys. J. Neurosci. 30, 8591–8601 10.1523/JNEUROSCI.0049-10.2010 20573905 PMC2942083

[B11] Bowman E. M. Aigner T. G. Richmond B. J. (1996). Neural signals in the monkey ventral striatum related to motivation for juice and cocaine rewards. J. Neurophysiol. 75, 1061–1073 8867118 10.1152/jn.1996.75.3.1061

[B12] Chiba T. Kayahara T. Nakano K. (2001). Efferent projections of infralimbic and prelimbic areas of the medial prefrontal cortex in the Japanese monkey, *Macaca fuscata*. Brain Res. 888, 83–101 10.1016/S0006-8993(00)03013-4 11146055

[B13] Collet C. Dittmar A. Vernet-Maury E. (1999). Programming or inhibiting action: evidence for differential autonomic nervous system response patterns. Int. J. Psychophysiol. 32, 261–276 10.1016/S0167-8760(99)00022-7 10437637

[B14] Dommett E. Coizet V. Blaha C. D. Martindale J. Lefebvre V. Walton N. Mayhew J. E. W. Overton P. G. Redgrave P. (2005). How visual stimuli activate dopaminergic neurons at short latency. Science 307, 1476–1479 10.1126/science.1107026 15746431

[B15] Doya K. (2008). Modulators of decision making. Nat. Neurosci. 11, 410–416 10.1038/nn2077 18368048

[B16] Flagel S. Clark J. Robinson T. Mayo L. Czuj A. Willuhn I. Akers C. Clinton S. Phillips P. E. M. Akil H. (2011). A selective role for dopamine in stimulus-reward learning. Nature 469, 53–57 10.1038/nature09588 21150898 PMC3058375

[B17] Gan J. O. Walton M. E. Phillips P. E. M. (2010). Dissociable cost and benefit encoding of future rewards by mesolimbic dopamine. Nat. Neurosci. 13, 25–27 10.1038/nn.2460 19904261 PMC2800310

[B18] Hazy T. E. Frank M. J. O'Reilly R. C. (2010). Neural mechanisms of acquired phasic dopamine responses in learning. Neurosci. Biobehav. Rev. 34, 701–720 10.1016/j.neubiorev.2009.11.019 19944716 PMC2839018

[B19] La Camera G. Richmond B. J. (2008). Modeling the violation of reward maximization and invariance in reinforcement schedules. PLoS Comput. Biol. 4:e1000131 10.1371/journal.pcbi.1000131 18688266 PMC2453237

[B20] Lebreton M. Jorge S. Michel V. Thirion B. Pessiglione M. (2009). An automatic valuation system in the human brain: evidence from functional neuroimaging. Neuron 64, 431–439 10.1016/j.neuron.2009.09.040 19914190

[B21] Matsumoto M. Hikosaka O. (2009). Two types of dopamine neuron distinctly convey positive and negative motivational signals. Nature 459, 837–841 10.1038/nature08028 19448610 PMC2739096

[B22] McGaughy J. Ross R. S. Eichenbaum H. (2008). Noradrenergic, but not cholinergic, deafferentation of prefrontal cortex impairs attentional set-shifting. Neuroscience 153, 63–71 10.1016/j.neuroscience.2008.01.064 18355972 PMC2615225

[B23] Minamimoto T. Hori Y. Kimura M. (2005). Complementary process to response bias in the centromedian nucleus of the thalamus. Science 308, 1798–1801 10.1126/science.1109154 15961671

[B24] Minamimoto T. Hori Y. Kimura M. (2009). Roles of the thalamic CM-PF complex-Basal ganglia circuit in externally driven rebias of action. Brain Res. Bull. 78, 75–79 10.1016/j.brainresbull.2008.08.013 18793702

[B25] Nomoto K. Schultz W. Watanabe T. Sakagami M. (2010). Temporally extended dopamine responses to perceptually demanding reward-predictive stimuli. J. Neurosci. 30, 10692–10702 10.1523/JNEUROSCI.4828-09.2010 20702700 PMC3297489

[B26] Okada K.-I. Toyama K. Inoue Y. Isa T. Kobayashi Y. (2009). Different pedunculopontine tegmental neurons signal predicted and actual task rewards. J. Neurosci. 29, 4858–4870 10.1523/JNEUROSCI.4415-08.2009 19369554 PMC6665332

[B27] Padoa-Schioppa C. Assad J. A. (2006). Neurons in the orbitofrontal cortex encode economic value. Nature 441, 223–226 10.1038/nature04676 16633341 PMC2630027

[B28] Pavlov I. (2003). Conditioned Reflexes. Mineola, NY: Dover Publications

[B28a] Pessiglione M. Seymour B. Flandin G. Dolan R. J. Frith C. D. (2006). Dopamine-dependent prediction errors underpin reward-seeking behaviour in humans. Nature 442, 1042–1045 10.1038/nature05051 16929307 PMC2636869

[B29] Phillips A. G. Ahn S. Howland J. G. (2003). Amygdalar control of the mesocorticolimbic dopamine system: parallel pathways to motivated behavior. Neurosci. Biobehav. Rev. 27, 543–554 10.1016/j.neubiorev.2003.09.002 14599435

[B30] Rajkowski J. Majczynski H. Clayton E. C. Aston-Jones G. (2004). Activation of monkey locus coeruleus neurons varies with difficulty and performance in a target detection task. J. Neurophysiol. 92, 361–371 10.1152/jn.00673.2003 15028743

[B31] Ravel S. Richmond B. J. (2006). Dopamine neuronal responses in monkeys performing visually cued reward schedules. Eur. J. Neurosci. 24, 277–290 10.1111/j.1460-9568.2006.04905.x 16882024

[B32] Redgrave P. Gurney K. (2006). The short-latency dopamine signal: a role in discovering novel actions? Nat. Rev. Neurosci. 7, 967–975 10.1038/nrn2022 17115078

[B33] Robbins T. W. Roberts A. C. (2007). Differential regulation of fronto-executive function by the monoamines and acetylcholine. Cereb. Cortex 17(Suppl. 1), i151–i160 10.1093/cercor/bhm066 17725997

[B34] Sara S. J. (2009). The locus coeruleus and noradrenergic modulation of cognition. Nat. Rev. Neurosci. 10, 211–223 10.1038/nrn2573 19190638

[B35] Schultz W. (2010). Dopamine signals for reward value and risk: basic and recent data. Behav. Brain Funct. 6, 24 10.1186/1744-9081-6-24 20416052 PMC2876988

[B36] Schultz W. Dayan P. Montague P. R. (1997). A neural substrate of prediction and reward. Science 275, 1593–1599 10.1126/science.275.5306.1593 9054347

[B37] Simmons J. M. Minamimoto T. Murray E. A. Richmond B. J. (2010). Selective ablations reveal that orbital and lateral prefrontal cortex play different roles in estimating predicted reward value. J. Neurosci. 30, 15878–15887 10.1523/JNEUROSCI.1802-10.2010 21106826 PMC3021956

[B38] Simmons J. M. Richmond B. J. (2008). Dynamic changes in representations of preceding and upcoming reward in monkey orbitofrontal cortex. Cereb. Cortex 18, 93–103 10.1093/cercor/bhm034 17434918

[B39] Slovin H. Abeles M. Vaadia E. Haalman I. Prut Y. Bergman H. (1999). Frontal cognitive impairments and saccadic deficits in low-dose MPTP-treated monkeys. J. Neurophysiol. 81, 858–874 10036286 10.1152/jn.1999.81.2.858

[B40] Ventura R. Morrone C. Puglisi-Allegra S. (2007). Prefrontal/accumbal catecholamine system determines motivational salience attribution to both reward- and aversion-related stimuli. Proc. Natl. Acad. Sci. U.S.A. 104, 5181–5186 10.1073/pnas.0610178104 17360372 PMC1820518

[B41] Wanat M. J. Kuhnen C. M. Phillips P. E. M. (2010). Delays conferred by escalating costs modulate dopamine release to rewards but not their predictors. J. Neurosci. 30, 12020–12027 10.1523/JNEUROSCI.2691-10.2010 20826665 PMC2946195

[B42] Yu A. J. Dayan P. (2005). Uncertainty, neuromodulation, and attention. Neuron 46, 681–692 10.1016/j.neuron.2005.04.026 15944135

